# Comparing age differences in cognition, personality, and political orientation across six online recruitment platforms

**DOI:** 10.3758/s13428-026-03027-8

**Published:** 2026-04-28

**Authors:** Michael S. Cohen, Karolina M. Lempert, David A. Wolk, Joseph W. Kable

**Affiliations:** 1 Department of Psychology, University of Pennsylvania, Philadelphia, PA 19104 USA; 2 Department of Psychology, University of Chicago, Chicago, IL USA; 3 Gordon F. Derner School of Psychology, Adelphi University, Garden City, NY USA; 4 Department of Neurology and Penn Memory Center, Hospital of the University of Pennsylvania, Philadelphia, PA USA

**Keywords:** Cognitive aging, Socioemotional aging, Personality, Online data collection, Methodology

## Abstract

Aging is typically associated with characteristic changes to cognitive function, personality, and political orientation. While online data collection for psychological research has greatly increased in frequency, there has been little systematic examination of whether online samples are appropriate for studying aging. Here, we examine whether typical age-related differences in cognitive function, personality, and political orientation are replicated in online samples. We measured cognitive performance using tests of vocabulary, processing speed, memory, and attention developed by the TestMyBrain Project; personality using the Big Five Inventory; and political orientation following the ANES survey format. At least 200 participants each were sampled from three crowdsourcing websites (Amazon MTurk, CloudResearch MTurk Toolkit, and Prolific) and three panel recruitment websites (Lucid, CloudResearch PrimePanels, and Qualtrics Panels), with age ranging from 18 to 92 years across platforms. On all six platforms, consistent with established findings, age was positively correlated with vocabulary performance and negatively correlated with processing speed. Additionally, in all six samples, consistent with prior studies, age was associated with higher Agreeableness, lower Neuroticism, and greater political conservatism. There were some differences between crowdsourced and panel samples, however. Performance on cognitive measures was broadly better for crowdsourced samples. The correlations between age and Openness and Extraversion differed between crowdsourced and panel samples, with trends in panel samples likely more comparable to the ground truth. Finally, MTurk produced some discrepant effects of age on cognition relative to other crowdsourced platforms. Beyond these differences, though, our results are broadly encouraging for the prospect of studying aging via online experiments.

**Supplementary Information:**

The online version contains supplementary material available at 10.3758/s13428-026-03027-8.

## Introduction

Over the past decade, researchers in the social sciences have increasingly used online data collection. Online data collection has several advantages over in-person data collection, including the ability to collect larger and more diverse samples, greater convenience for researchers and participants, and reduced costs. The COVID-19 pandemic, which greatly reduced opportunities to collect in-person data safely, further accelerated this shift. Still, concerns have been raised about the representativeness of older adults who participate in online studies (e.g., Ogletree & Katz, 2021). There is a need for more systematic comparisons of performance across age groups among participants recruited online to test whether online recruitment yields age differences comparable to those from more traditional recruitment methods.

One major distinction in online recruitment is between crowdsourcing and panel services. On crowdsourcing platforms, such as Amazon MTurk, CloudResearch MTurk Toolkit, and Prolific, participants are presented with a list of available studies. The details of each study are provided, including the tasks participants will complete, the required time commitment, and the compensation amount (in cash). People can then choose to participate in a specific study. Online panels instead build on the approach of market research panels that predated online data collection. These services differ from crowdsourcing platforms in that they approach participants who are already part of a panel to participate in a specific study. The amount and type of participant compensation is controlled by the panel service. Panels typically have access to a much larger and more diverse sample of participants than crowdsourcing sites, which can allow for greater representativeness, more naivete with regard to experimental manipulations, and more precise targeting (Chandler et al., 2019; Moss et al., 2023). Panels have notable limitations, however. For instance, panel services often limit studies to approximately 20 min or less. Additionally, panel services work with many panel providers, who bid on each study, meaning that multiple sources can be aggregated for a single study and also that different studies with the same panel service do not necessarily draw upon the same sources (Moss et al., 2023). Three of the most widely used online panel recruitment platforms are Lucid (which in late 2021 was absorbed into another company, Cint), CloudResearch’s Prime Panels, and Qualtrics Panels.

### Validation of paid online recruitment platforms

Putting aside the question of age differences, many studies have examined other aspects of the validity of online data collection since it first became widespread. Several studies have focused on Amazon’s Mechanical Turk (“MTurk”), the first platform to be widely used for recruiting research participants. For instance, Berinsky et al. (2012) replicated characteristic biases in judgment and decision-making tasks in MTurk samples. They also found that MTurk samples are diverse demographically, but do lean more liberal politically, and, at that time, had very few older adults. Other studies have examined whether presentation or response time effects can be reliably measured online. Here, Crump et al. (2013) found that tasks manipulating stimulus presentation times or measuring response times show comparable results in MTurk samples as in traditional samples, with limited exceptions.

In more recent years, alternative platforms have become available that address one of the limitations of MTurk, which is that Amazon does little screening of MTurk workers. The primary mechanism by which workers are screened is rejection rate on prior tasks. However, academic researchers are typically expected to compensate all participants who complete a study regardless of data quality; thus, MTurk accounts generating poor quality data (e.g., bots or inattentive participants) may still have a low rejection rate. Prolific and CloudResearch aim to address this concern. Prolific has its own participant pool, while the CloudResearch MTurk Toolkit (“CR Toolkit”) applies quality filters to the existing MTurk participant pool. (Note that since the data reported here were collected, CloudResearch has begun to phase out CR Toolkit in favor of a new platform, Connect, which, like Prolific, maintains its own participant pool.) The steps implemented by both Prolific and CR Toolkit appear to improve data quality relative to MTurk (e.g., Douglas et al., 2023; Hauser et al., 2022; Peer et al., 2021; Stagnaro et al., 2024). Broadly, Prolific and CR Toolkit participants show better performance on a range of measures, including effect sizes on established tasks, attention check pass rate, consistency of survey responses, and compliance with instructions.

Several studies in recent years have compared online platforms on data quality and representativeness. Lucid panels were found to be more representative on demographic and personality measures than MTurk, while on both platforms, classic judgment and decision-making findings replicated (Coppock & McClellan, 2019). Chandler et al. (2019) similarly found that classic behavioral effects replicated in a Prime Panels sample, which was also closer to representative demographically than MTurk and had less prior exposure to common research questionnaires. Peer et al. (2021) found little difference in data quality between MTurk and Qualtrics Panels. Douglas et al. (2023) found, however, that Qualtrics Panels participants performed more poorly on attention check measures than those recruited via CR Toolkit or Prolific, but somewhat better than those recruited from MTurk. Stagnaro et al. (2024) recently assessed many platforms on both data quality and representativeness, generally finding a tradeoff between the two. Specifically, data collected via Prolific and CloudResearch (both Connect and CR Toolkit) showed higher data quality but lower representativeness, while Lucid showed the reverse. MTurk was an exception, showing both low data quality and poor representativeness.

Another consideration is that data quality on different platforms may change over time. For instance, contrary to more recent work indicating better data quality on Prolific and CR Toolkit, an earlier study by Peer et al. (2017) found no difference in data quality between Prolific and MTurk. There is also evidence for discrete changes in data quality online. For example, some researchers have reported reductions in attentiveness and cognitive performance on both MTurk (Arechar & Rand, 2021) and Lucid (Ternovski & Orr, 2022) when comparing data collected in early 2020 before the COVID pandemic to data collected after the onset of the pandemic through mid-2021. Other researchers observed a notable increase in the frequency of low-quality responses on MTurk beginning in mid-2018 (e.g., Chmielewski & Kucker, 2019; Kennedy et al., 2020), which affected the validity of results (e.g., Ophir et al., 2019). Demographic distortions in the Prolific participant pool emerged after a blogger promoted the service in July 2021 as a money-making opportunity to her audience of mostly young women (Charalambides, 2021). Given these examples, periodic reassessment of data quality on online platforms is advisable.

### Testing older adults online

The above studies, however, have not addressed whether samples recruited online reveal the same characteristic patterns of age differences in cognition, personality and political orientation that have been observed in traditionally recruited samples. On one hand, older adults may show worse performance on cognitive tests self-administered at home via a computer or tablet than when a researcher or clinician administers a test in an office. Causes here could include increased cognitive load due to lack of familiarity with the technology, stereotype threat related to use of technology, or other factors that impair performance in older but not young adults. On the other hand, there are reasons why older adults recruited online may perform better than traditional samples. First, it is easy to imagine that older adults who volunteer to participate via online crowdsourcing services are drawn from a subset of the population with higher socioeconomic status and/or cognitive abilities than the typical young adult on the same platforms. Older adult samples in cognitive aging studies are traditionally recruited using methods such as in-person contacts, newsletters, and doctors’ offices, which may achieve a relatively more representative sample of the older adult population. The context of being tested in a laboratory or medical office may also activate negative age-related stereotypes about memory and/or brain health, thereby impairing cognitive performance in older adults (Hess et al., 2003). Online testing, which would usually be done at home, may remove this threat. Because online testing of cognitive performance could vary in either direction from traditional samples in older adults, prior findings that online data is valid across the population at large do not address the issue of whether basic differences between older and young adults replicate when both age groups are recruited online.

Several studies have examined the reliability of self-administered online tests, relative to cognitive tests administered in-person, in older adults (e.g., Assmann et al., 2016; Backx et al., 2020; Cyr et al., 2021; Eve & de Jager, 2014; Feenstra et al., 2018). These studies focus on important questions about possible variability in performance due to reduced technical literacy in older adults and find that performance on computerized tests is generally in alignment with more traditional methods of administration. Other studies have examined age differences online, finding consistency between normative data and large participant samples recruited online. Tasks that have been examined in this manner include vocabulary, digit symbol coding, and working memory (Hartshorne & Germine, 2015), sustained attention (Fortenbaugh et al., 2015), as well as working memory span, feature binding, and prospective memory (Logie & Maylor, 2009). In these studies, however, participants were not financially compensated. Thus, these studies do not address possible biases in samples from paid recruitment platforms.

To our knowledge, there are three studies that have examined cognitive performance in older adults recruited through paid online platforms. One study compared an MTurk sample with older adults recruited through a traditional longitudinal study (Ogletree & Katz, 2021). Here, the MTurk group showed better performance on analogical reasoning and verbal fluency tasks, suggesting that older adults who sign up for online studies may be a higher-functioning group than the general population. At the same time, Bui et al. (2015) reported typical age-related declines in processing speed, and expected modulations of this effect in three sets of participants recruited from MTurk. The Bui et al. study used a similar approach to the present work but was more limited in the scope of tasks addressed and in examining only a single platform. Finally, Greene and Naveh-Benjamin (2022) showed that, for one specific memory task, age effects on performance were comparable between a sample collected via Prolific during the COVID-19 pandemic and an in-lab sample collected pre-pandemic.

None of these prior studies provides a systematic evaluation of age differences on measures of cognition, personality, and political orientation across different online recruitment platforms. The present study provides such an evaluation, examining age differences across six different paid online recruitment platforms, including both crowdsourced and panel populations, on a variety of measures, including cognitive performance, personality, and political preferences. We therefore aim to provide important validation for researchers who wish to examine aging via online studies in future work.

### Measures

We chose measures that have demonstrated reliable and reproducible differences between young and older adults in order to determine whether those effects replicate across six online platforms.

### Cognitive performance

To assess cognitive performance, we selected four tasks: vocabulary, digit symbol coding, paired associate memory, and sustained attention. The vocabulary and digit symbol coding tasks used here have been validated in a large online sample as showing comparable age-related differences as the Wechsler Adult Intelligence Scale (WAIS) intelligence test norms. The vocabulary measure shows a characteristic increase in performance with age, while the digit symbol coding measure shows a characteristic decrease in performance with age (Hartshorne & Germine, 2015). We also examined paired associate memory, a type of memory test that typically shows age-related decreases (e.g., Old & Naveh-Benjamin, 2008). The final task, the gradual-onset continuous performance task (gradCPT), measures individual differences in sustained attention. Fortenbaugh et al. (2015) found that overall accuracy in this task, represented by d’, does not show a linear main effect across the adult life span; instead, it gradually increases through young adulthood to a peak in middle age (early 40 s), then gradually decreases. Strategy, a second measure quantified using response criterion, shows a negative linear effect with age, with older participants becoming more cautious in responding. While age differences on the gradCPT are not as straightforward as for the other cognitive measures, we chose it to assess whether there are age-related differences in the attentiveness of participants recruited from different paid online participant pools.

### Personality

We chose the Big Five Inventory (BFI) to assess personality. Two cross-sectional studies administered this measure to very large samples of participants who signed up voluntarily for an online personality test and found a clear pattern of age differences (Soto et al., 2011; Srivastava et al., 2003). Both studies draw on data from the Gosling-Potter Internet Personality Project (GPIPP), but from different time windows: Srivastava et al. (2003) included data collected between 1998 and 2000, while Soto et al. (2011) reported data collected from 2003 to 2009. Both studies showed positive associations with age for Agreeableness and Conscientiousness, and a negative association between age and Neuroticism, from young adulthood to late middle age (age 60 or 65). Extraversion did not show a linear effect with age in either study. Openness is the only factor to show different effects in the two studies, with Srivastava et al. (2003) finding a decrease, while Soto et al. (2011) found an increase with age across the adult life span. Other studies of personality and age, using traditionally recruited samples, have typically observed similar positive associations between age and Agreeableness and negative associations between age and Neuroticism. There are some differences from the GPIPP online samples, however, as traditional studies have consistently found age-related decreases in Extraversion and Openness, as well as a less-consistent age-related decrease in Conscientiousness, especially past age 75 (Donnellan & Lucas, 2008; Graham et al., 2020; Roberts et al., 2006).

### Political orientation

To assess political ideology and political party affiliation, we adopted the question format of the American National Election Studies (ANES), which provides data on these questions going back to 1972. One study used the ANES to examine changes over time within and between generations (Fisher, 2020). This analysis showed that beginning in 2008, through at least 2016, the most recent dataset included in that study, each younger generation was more liberal than the one preceding it. There was some evidence for such a relationship in prior decades as well, though less consistently. This analysis also showed age-related increases in conservatism and Republican Presidential vote share over time within each generation. Other data have similarly suggested increased conservatism with age within generations among Americans (e.g., Peltzman, 2019; Peterson et al., 2020) and in other countries as well (e.g., Tilley & Evans, 2014). Thus, there is good reason to believe that, at the time the data for the present study were collected, age would be associated with greater ideological conservatism and Republican party identity. 

## Method

### Participants

Complete datasets (i.e., participants passing all attention checks and completing all core measures) were obtained from 1829 participants across six recruitment platforms: three crowdsourcing sites (Amazon MTurk, CloudResearch MTurk Toolkit (CR Toolkit), and Prolific) and three panel recruitment platforms (Lucid, Prime Panels, and Qualtrics Panels). Data were not recorded from participants who failed the simple attention checks described below or who did not complete all parts of the study. Of the participants who completed all parts of the study, 71 were excluded: 56 for entering an age and birth year that did not match, 13 for not providing enough information to assess their level of education, and two for completing the full study twice through both MTurk and CR Toolkit. For participants who completed one or more parts of the study more than once, only the first responses were included for analysis. The final sample comprised 1758 participants.

Participation was limited to U.S. residents who were using a desktop or laptop computer. For MTurk and CR Toolkit, an 85% Amazon MTurk approval threshold was used. For CR Toolkit, we additionally applied the default “CloudResearch Approved Participants” filter. All crowdsourced participants were paid $4 each, while the platform determined compensation for panel participants. The intended sample was 210 participants per platform, with an even distribution across the adult age range; Table 1 shows the actual distribution of participants by age. The total sample was 52.1% female, 47.5% male, and 0.4% other/non-binary/did not report gender (see Table 1 for gender distribution by platform). Data were collected in spring and early summer of 2021, providing an important reference point for assessing the quality of online data collected at a specific time point relative to the COVID-19 pandemic (see Table 1 for the precise dates of data collection for each platform). Costs for each platform are reported in Supplemental Table 1.

**Table 1:** Demographic information by platform

Platform		MTurk		CR Toolkit		Prolific		Lucid		Prime Panels		Qualtrics Panels	
		*n*	%	*n*	%	*n*	%	*n*	%	*n*	%	*n*	%
n by age group	Age 18–29	47	26.9	49	25.0	45	22.3	23	4.4	20	9.5	28	5.4
	Age 30–39	29	16.6	34	17.3	35	17.3	61	13.4	30	14.2	30	5.8
	Age 40–49	13	7.4	26	13.3	29	14.4	60	13.2	21	10.0	33	6.4
	Age 50–59	29	16.6	22	11.2	39	19.3	51	11.2	36	17.1	44	8.5
	Age 60–69	47	26.9	39	19.9	35	17.3	120	26.3	36	17.1	180	34.7
	Age 70–79	10	5.7	24	12.2	18	8.9	113	24.8	33	15.6	172	33.2
	Age 80 +	0	0	2	1.0	1	0.5	28	6.1	35	16.6	31	6.0
n by gender	Female	102	58.3	98	50.0	106	52.5	188	41.2	115	54.5	307	59.3
	Male	72	41.1	96	49.0	94	46.5	268	58.8	96	45.5	209	40.3
	Other Gender / No response	1	0.6	2	1.0	2	1.0	0	0	0	0	2	0.4
n by education level	Did not finish high school	0	0	0	0	1	0.5	4	0.9	3	1.4	11	2.1
	High school	21	12.0	31	15.8	19	9.4	70	15.4	36	17.1	103	19.9
	Some college	33	18.9	52	26.5	40	19.8	90	19.7	36	17.1	108	20.8
	Associate’s degree	17	9.7	11	5.6	15	7.4	51	11.2	21	10.0	56	10.8
	Bachelor’s degree	69	39.4	83	42.3	73	36.1	125	27.4	62	29.4	141	27.2
	Master’s degree	32	18.3	15	7.7	41	20.3	87	19.1	45	21.3	85	16.4
	Doctoral degree	2	1.1	3	1.5	11	5.4	26	5.7	8	3.8	12	2.3
	Other	1	0.6	1	0.5	2	1.0	3	0.7	0	0	2	0.4
Mean (SD) age in years by platform		46.6 (16.9)		46.8 (18.2)		46.8 (17.2)		58.2 (16.7)		57.6 (19.3)		63.0 (15.3)	
Dates tested		4/27/21–4/28/21		4/27/21–4/28/21		4/27/21–4/28/21		4/27/21–4/29/21		6/4/21–6/13/21		7/1/21–7/5/21	

It should be noted that although our recruitment goals were broadly consistent across platforms, i.e., an even distribution of participants across the adult lifespan, the precise age distributions that we were allowed to request and that the platforms successfully provided were not uniform. For the panel platforms (Lucid, Prime Panels, and Qualtrics), we requested 30 participants in each of seven age brackets: 18–29, 30–39, 40–49, 50–59, 60–69, 70–79, 80 + . Samples from Lucid and Qualtrics were notably larger than what we requested, particularly in the older age brackets; these data were supplied at no additional charge, and we elected to retain all valid data. The crowdsourced platforms had fewer available participants in the oldest age brackets and allowed less specificity in requesting older participants, so we approximated this distribution as closely as possible. For MTurk, 70 participants were requested in each of three age brackets (18–35, 35–55, 55 +), which was the maximum level of control allowed. The final MTurk sample was smaller than those on other platforms because many exclusions after data collection, due to data quality issues, were from this sample. For CR Toolkit, the requested sample included 30 participants in each of five age brackets (18–24, 25–34, 35–44, 45–54, 55–64). Initially, we requested an additional 20 participants age 65–70 and 40 participants age 71 or older, but due to failure to complete the quota for participants aged 71 + on the first attempt, we relaxed this specification to age 65 + for the final 15 older participants. For Prolific, the requested sample was 25 participants age 18–24, 35 participants in each of the next five age brackets (25–34, 35–44, 45–54, 55–64, 65–74), and ten participants age 75 + .

### Procedure

In order to begin the study, participants had to provide informed consent, and to successfully complete three simple attention checks (selecting an odd number from three choices, selecting a picture of a cat from three choices, and an automated Captcha). They then completed the 44-item Big Five Inventory (BFI; John & Srivastava, 1999) and provided their year of birth. Participants then completed cognitive tasks on Testmybrain.org in the following order: Vocabulary, Paired Associate Memory encoding, Digit Symbol Coding, Paired Associate Memory test, and gradual onset Continuous Performance Test (gradCPT). Vocabulary and Paired Associate Memory were scored as the proportion of items correct. Digit symbol coding was scored as the number of items correct. GradCPT provided measures of accuracy (d’) and criterion. Participants had the opportunity to view their scores for all four tests after they were completed. Finally, participants provided demographic and other information, including age (compared against year of birth as an attention check), gender, race/ethnicity, education, income, household size, ZIP code, political party, and ideology. Education level was collected categorically, which we later converted to years of education (see Supplemental Table 2). Years of education were manually coded for nine participants who responded “other” to education level and provided additional information.

**Table 2:** Effects of age on cognitive performance measures within each platform, controlling for level of education

	MTurk			CR toolkit			Prolific				
	*b*	*t*	Corrected *p*	*b*	*t*	Corrected *p*	*b*	t			Corrected p
Vocabulary	**0.0072**	**7.42**	< .001***	**0.0032**	**5.58**	< .001***	**0.0042**	**5.53**			< .001***
Digit Symbol Coding	**– 0.3098**	**– 5.28**	< .001***	**– 0.5189**	**– 12.83**	< .001***	**– 0.4802**	**– 10.08**			< .001***
Paired Assoc Memory	– 0.0003	– 0.33	.74	– 0.0012	– 1.79	.15	**– 0.0024**	**– 3.43**			.002**
GradCPT d’	**0.0197**	**4.24**	< .001***	0.0015	0.45	1	0.0016	0.44			1
GradCPT criterion	– 0.0027	– 1.12	.53	**– 0.0064**	**– 4.27**	< .001***	**– 0.0053**	**– 2.75**			.033*
	Lucid			Prime panels			Qualtrics panels				
	*b*	*t*	Corrected *p*	*b*	*t*	Corrected p	*b*	t			Corrected *p*
Vocabulary	**0.0065**	**10.73**	< .001***	**0.0043**	**7.10**	< .001***	**0.0062**		**11.42**	< .001***	
Digit Symbol Coding	**– 0.4270**	**– 10.65**	< .001***	**– 0.4860**	**– 9.25**	< .001***	**– 0.4066**		**– 9.79**	< .001***	
Paired Assoc Memory	**– 0.0019**	**– 5.75**	< .001***	**– 0.0021**	**– 5.73**	< .001***	**– 0.0015**		**– 4.74**	< .001***	
GradCPT d’	0.0023	0.87	1	0.0002	0.58	1	0.0064		2.55	.056 ~	
GradCPT criterion	– 0.0037	– 2.16	.12	– 0.0037	– 1.82	.21	0.0011		0.64	.53	

Reported *p* values for each measure include a Bonferroni–Holm correction for multiple comparisons across the six platforms

Political party and ideology were collected in the format of the American National Election Studies (ANES). A single seven-point scale was used for political ideology, ranging from “Extremely Liberal” to “Extremely Conservative”. For the political party, responses were converted to a seven-point scale based on two questions. First, participants were asked whether they think of themselves as Republican, Democrat, Independent, Other, or No Preference. If they chose “Republican” or “Democrat”, a second question asked whether they are “Strong” or “Not Very Strong” in that affiliation; these responses form the ends of the seven-point scale. If they chose another response, they were then asked whether they are closer to the Republican party, the Democratic party, or neither; these responses form the three intermediate points of the seven-point scale.

Comparison data for cognitive measures were provided by the Many Brains Project, corresponding to people of all ages and locations who voluntarily took the same tests for no compensation at Testmybrain.org. We limited the comparison data to participants over the age of 18, to correspond with our sample, yielding the following sample sizes: Vocabulary (*n* = 33,321), Digit Symbol Coding (*n* = 6862), Paired Associate Memory (*n* = 9085), GradCPT (*n* = 19,023). A similar large comparison sample of unpaid volunteers were obtained for personality measures from the GPIPP dataset, incorporating all data up to 03/25/2015. We included participants who reported living in the United States and ranging in age from 18 to 85, encompassing 2,669,696 data points. Finally, normative data on political ideology and partisan orientation were taken from the 2020 ANES dataset available via the https://electionstudies.org website. As each comparison dataset used somewhat different categories for level of education, the coding for years of education differed slightly for each dataset; conversions from categories to years for each dataset are reported in Supplemental Table 2.

We first examined regressions predicting outcome measures that did not account for differences between platforms. These regressions were run separately on each of the six tested platforms, as well as on all data combined across platforms. For each of these regressions, the predictor variables were participant age and years of education.

We then examined models that included data from all 6 platforms, with regressors of age (centered on 48 years of age, as the midpoint between the onset of adulthood at 18 years and typical U.S. life expectancy of about 78 years), platform type (a dummy regressor valued at 0 for crowdsourced platforms and 1 for panels), and the interaction between platform type and age, with years of education as a control variable. Centering age at 48 years, for these regressions and the others described below, allows us to interpret main effects modeled alongside an interaction term as an effect of, e.g., platform type, at middle age (48 years old). Main effects of platform type indicate differences in cognition/personality/political orientation between crowdsourced and panel platforms, while interactions reflect that the effects of age differ by platform type.

Then, to determine whether models adding terms for main effects of specific platform and interactions between platform and age were merited, we used ANOVA to compare the proportion of variance explained by models with only terms for platform type to models with additional terms for each specific platform. Reference conditions in these latter models were arbitrarily chosen to be MTurk for crowdsourced data and Lucid for panels. The models included terms for main effects of age (centered on 48 years old) and education, two dummy regressors representing main effects of platform (CR Toolkit and Prolific for crowdsourced data, Prime Panels and Qualtrics for panel data), and two regressors representing interactions between age and each dummy-coded platform. Results from models accounting for platform effects were reported only when the ANOVA showed that they explained additional variance.

Finally, we compared the data from each individual platform to the larger unpaid volunteer samples from other sources, described above. Here, comparison data for a given measure were concatenated with the data that we collected from all six platforms. Main effects of age (centered on 48 years old) and years of education were modeled, as were six dummy regressors coding main effects for each platform, and six regressors coding the interaction between each platform and age.

Regression models were generally fit using the Python smf.ols function, part of the statsmodels module. For regressions that included ANES data on political orientation, we used weighted least squares regression via the smf.wls function. This allowed us to incorporate the weights provided in the ANES dataset to better approximate the actual U.S. population; the data that we collected were all given a weight of 1. For plots showing data from each platform, our data were combined with the comparison data, and variance based on years of education was removed by running a regression with a single predictor variable of education for each outcome measure. The mean fitted value from each regression was then added to the residuals in order to match the original scale. Linear regression lines indicating the effects of age on these residuals were computed separately for the comparison data and for the data that we collected within each platform.

## Results

### Cognitive performance measures

**Fig. 1:**
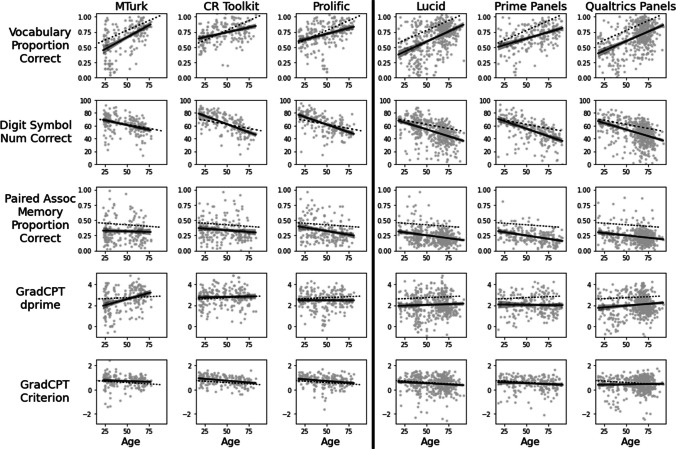
Relationships between age and cognitive measures by platform, controlling for level of education, with solid lines showing linear regression effects and 95% confidence intervals within each platform. Dashed lines represent regression effects from Testmybrain’s large unpaid volunteer samples, controlling for level of education

Examining each platform individually, we found the expected age differences in vocabulary and processing speed on all platforms, and the expected age differences in memory and sustained attention on most platforms. Figure 1 shows the relationship between age and each cognitive performance measure on each platform while controlling for level of education. Statistics for the effects of age when regressing age and education on each outcome measure within each platform are reported in Table 2. Within each platform, age was positively associated with vocabulary performance and negatively associated with digit symbol coding performance, as expected from prior work. Age was also a negative predictor of paired associate memory, as expected based on prior work, on four of the six platforms, with null results on MTurk and CR Toolkit. GradCPT showed expected effects of age on CR Toolkit and Prolific, i.e., no linear effect of age on d’ and a negative effect of age on criterion. A different pattern was apparent on MTurk, which showed a strong positive correlation between age and d’ and no age differences in criterion, as well as Qualtrics Panels, which showed a marginal positive effect of age on d’ with no effect of age on criterion. Effects of both d’ and criterion were null on Lucid and Prime Panels.

Regressions combining data across all platforms, with only age and education as regressors, showed the expected age-related increase in vocabulary (*b* = 0.0046, *t* = 17.27, *p* < .001), age-related decrease in digit-symbol coding (*b* = – 0.5005, *t* = – 27.31, *p* < .001), and age-related decrease in paired-associate memory (*b* = – 0.0023, *t* = – 12.42, *p* < .001). The gradCPT showed no effect of age on d’ (*b* = – 0.0009, *t* = – 0.72, *p* = 0.47), as expected, and showed the expected decrease in criterion with age (*b* = – 0.0049, *t* = – 6.68, *p* < .001).

### Comparisons across platforms

Comparing platform type (crowdsourced vs. panels) at middle age, panels showed lower overall performance than crowdsourced samples on vocabulary (*b* = – 0.1088, *t* = – 10.12, *p* < .001), digit symbol coding (*b* = – 7.3175, *t* = – 9.78, *p* < .001), paired associate memory (*b* = – 0.0726, *t* = – 9.55, *p* < 0.001), and gradCPT d’ (*b* = – 0.6028, *t* = – 11.81, *p* < .001), as well as a lower (more cautious) gradCPT criterion (*b* = – 0.2268, *t* = – 7.44, *p* < .001). There were no significant interactions between platform type and age for any of the cognitive measures, though marginal trends were present for vocabulary (*b* = 0.0011, *t* = 1.90, *p* = .058), reflecting a trend towards a larger increase with age for panels, and gradCPT criterion (*b* = 0.0030, *t* = 1.86, *p* = .063), indicating that crowdsourced samples showed a larger decrease with age in the tendency to respond impulsively.

Within crowdsourced platforms, model-comparison ANOVAs indicated platform-specific effects in vocabulary (*F*(4, 566) = 9.92, *p* < .001), digit symbol coding (*F*(4, 566) = 3.74, *p* = .005), and gradCPT d’ (*F*(4, 566) = 5.95, *p* < .001), but not paired associate memory (*F*(4, 566) = 1.46, *p* = .21) or gradCPT criterion (*F*(4, 566) < 1). These platform-specific effects reflected a different pattern in MTurk participants compared to CR Toolkit and Prolific. The CR Toolkit sample showed better overall performance than MTurk on vocabulary (*b* = 0.0823, *t* = 4.30, *p* < .001) and gradCPT d’ (*b* = 0.2174, *t* = 2.26, *p* = .024) at middle age, with an additional marginal positive effect on digit symbol coding (*b* = 2.1602, *t* = 1.78, *p* = .075). The Prolific sample showed better overall performance than MTurk on vocabulary (*b* = 0.0724, *t* = 3.81, *p* < .001), but not on any other cognitive measure (all |*t*|< 1). Compared to MTurk, CR Toolkit and Prolific also displayed less of an increase in vocabulary performance with age (CR Toolkit: *b* = – 0.0041, *t* = – 3.72, *p* < .001; Prolific: *b* = – 0.0033, *t* = – 2.93, *p* = .004), a greater decrease in digit symbol coding with age (CR Toolkit: *b* = – 0.2186, *t* = – 3.15, *p* = .002; Prolific: *b* = – 0.1857, *t* = – 2.61, *p* = .009), and a less positive effect of age on gradCPT d’ (CR Toolkit: *b* = – 0.0190, *t* = – 3.45, *p* = .001; Prolific: *b* = – 0.0198, *t* = – 3.51, *p* < .001). Prolific also showed a greater decline with age in paired associate memory than MTurk (*b* = – 0.0022, *t* = – 2.06, *p* = .040).

Within panels, model-comparison ANOVAs only indicated platform-specific effects for vocabulary (*F*(4, 1178) = 2.53, *p* = .039). There were no differences between panel platforms for any of the other accuracy-based measures (all *F* < 1), nor for gradCPT criterion (*F*(4, 1178) = 1.40, *p* = .23). For vocabulary, Prime Panels showed higher performance than Lucid at middle age, as indicated by a significant main effect (*b* = 0.0515, *t* = 2.77, *p* = .006), as well as showing less of an increase in vocabulary performance with age (*b* = – 0.0021, *t* = – 2.30, *p* = .022). There were no differences between Qualtrics and Lucid in vocabulary performance or the effect of age on vocabulary (all |*t*|< 1).

### Comparisons with data from TestMyBrain

We compared the data from each of the six platforms to the comparison data provided by TestMyBrain from large online samples of unpaid volunteers (Fig. 1, dashed lines). Detailed results and statistics are reported in the Supplement. Table 3 shows the direction of any overall differences between each platform and the comparison data at middle age, and the direction of any differences in the effect of age between each platform and the comparison data.

**Table 3:** Direction of effects relative to TestMyBrain comparison samples (marginal effects are in parentheses)

	MTurk		CR Tookit		Prolific		Lucid		Prime Panels		Qualtrics Panels	
	Main Effect	Age	Main Effect	Age	Main Effect	Age	Main Effect	Age	Main Effect	Age	Main Effect	Age
Vocabulary	↓	—	↓	↓	↓	↓	↓	—	↓	↓	↓	—
Digit Symbol	—	↑	↑	↓	—	↓	↓	—	↓	↓	↓	—
Paired Assoc Memory	↓	—	↓	—	↓	—	↓	—	↓	—	↓	—
GradCPT d’	↓	↑	—	—	↓	—	↓	—	↓	(↓)	↓	—
GradCPT criterion	↑	(↑)	↑	—	↑	—	↓	(↑)	—	(↑)	↓	↑

*Up arrows* for main effects indicate better performance than the comparison group at middle age, while *down arrows* indicate poorer performance. Similarly, for age effects, *up arrows* indicate more a positive relationship between age and performance for a given platform relative to the comparison group, while *down arrows* indicate the reverse

For two of the five cognitive measures (vocabulary and paired associate memory), main effects indicated that all six platforms showed worse performance than the comparison data at middle age (48 years). The three panel platforms (Lucid, Prime Panels, Qualtrics Panels) also showed lower performance compared to the comparison data for digit symbol coding and sustained attention (gradCPT accuracy). Effects were less consistent across the crowdsourced platforms, as MTurk and Prolific were both comparable to the comparison group in digit symbol coding but worse on gradCPT accuracy, while CR Toolkit was comparable to the comparison group on gradCPT accuracy and better than the comparison group on digit symbol coding. Finally, participants on all crowdsourced platforms showed a less cautious (higher) gradCPT criterion relative to the comparison data at middle age, while the panels all showed at least a trend towards a more cautious (lower) gradCPT criterion than the comparison data at middle age.

Differences in the effects of age broadly do not show a consistent pattern, though on the two measures where the strongest age effects would be expected, CR Toolkit, Prolific, and Prime Panels all show a more negative effect of age, relative to the comparison data—that is, a weaker than expected increase with age for vocabulary, and a stronger than expected decrease with age for digit symbol coding.

### Personality measures

**Fig. 2:**
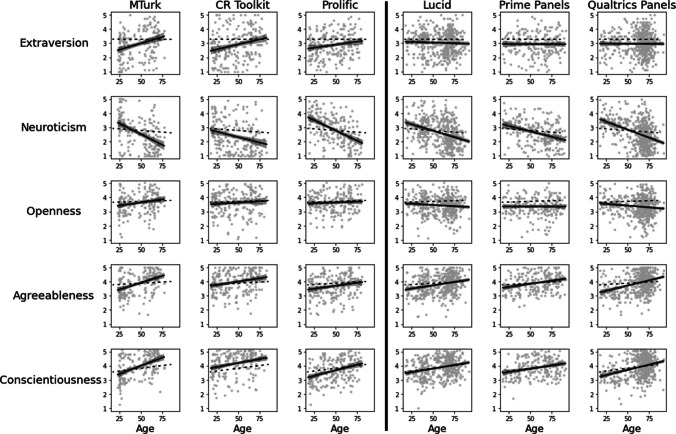
Relationships between age and personality measures by platform, controlling for level of education, with *solid lines* showing linear regression effects and 95% confidence intervals shown within each platform. *Dashed lines* represent regression effects from the large GPIPP personality dataset comparison sample, controlling for the level of education

Within each platform, the effects of age on personality were largely as expected, though with notable differences between platforms for Extraversion and Openness (Fig. 2; Table 4). Specifically, within each of the six recruitment platforms, age reliably predicted personality scores in the positive direction for Agreeableness and Conscientiousness, and in the negative direction for Neuroticism. Extraversion correlated positively with age on two of the three crowdsourcing platforms, MTurk and CR Toolkit, but showed no correlation with age on the three panel platforms or Prolific. Correlations with Openness were inconsistent across platforms—an age-related decrease on Qualtrics Panels, a marginal age-related increase on MTurk, and null effects on other platforms.

**Table 4:** Effects of age on personality measures within each platform, controlling for level of education

	MTurk			CR Toolkit			Prolific		
	*b*	*t*	Corrected *p*	*b*	*t*	Corrected *p*	*b*	*t*	Corrected *p*
Extraversion	**0.0196**	**4.49**	< .001***	**0.0137**	**3.30**	.006**	0.0076	2.18	.12
Neuroticism	**– 0.0300**	**– 6.79**	< .001***	**– 0.0152**	**– 3.71**	< .001***	**– 0.0285**	**– 8.30**	< .001***
Openness	0.0081	2.46	.075 ~	0.0033	1.03	.92	0.0020	0.71	.95
Agreeableness	**0.0190**	**5.90**	< .001***	**0.0093**	**3.25**	.003**	**0.0083**	**2.67**	.008**
Conscientiousness	**0.0209**	**6.34**	< .001***	**0.0115**	**3.91**	< .001***	**0.0163**	**5.32**	< .001***
	Lucid			Prime Panels			Qualtrics Panels		
	*b*	*t*	Corrected *p*	*b*	*t*	Corrected *p*	*b*	t	Corrected *p*
Extraversion	– 0.0016	– 0.74	1	– 0.0005	– 0.20	1	– 0.0001	– 0.06	1
Neuroticism	**– 0.0182**	**– 7.89**	< .001***	**– 0.0155**	**– 5.39**	< .001***	**– 0.0226**	**– 9.71**	< .001***
Openness	– 0.0032	– 1.90	.23	– 0.0003	– 0.14	.95	**– 0.0051**	**– 2.69**	.044*
Agreeableness	**0.0093**	**5.34**	< .001***	**0.0091**	**4.21**	< .001***	**0.0147**	**8.20**	< .001***
Conscientiousness	**0.0099**	**5.69**	< .001***	**0.0095**	**4.17**	< .001***	**0.0146**	**7.65**	< .001***

Reported *p* values for each measure include a Bonferroni–Holm correction for multiple comparisons across the six platforms

Combining across all platforms, we found expected main effects of age; older participants showed decreased Neuroticism (*b* = – 0.0185, *t* = – 16.14, *p* < .001), increased Agreeableness (*b* = 0.0104, *t* = 11.93, *p* < .001), and increased Conscientiousness (*b* = 0.0112, *t* = 12.39, *p* < .001). We also observed increased Extraversion with age (*b* = 0.0043, *t* = 3.89, *p* < .001) and decreased Openness with age (*b* = – 0.0027, *t* = – 3.02, *p* = .003), though as noted above and explored more below, these two effects were not consistent between different platforms in our dataset.

### Comparisons across platforms

Comparing between platform types at middle age (48 years), panel participants reported higher Extraversion (*b* = 0.0940, *t* = 2.03, *p* = .042), higher Neuroticism (*b* = 0.2260, *t* = 4.73, *p* < .001), lower Openness (*b* = – 0.1992, *t* = – 5.35, *p* < .001), lower Agreeableness (*b* = – 0.1163, *t* = – 3.19, *p* = .001), and lower Conscientiousness (*b* = – 0.1826, *t* = – 4.84, *p* < .001) compared to crowdsourced samples. There were also interactions between platform type and age for Extraversion (*b* = – 0.0141, *t* = – 5.70, *p* < .001), Neuroticism (*b* = 0.0053, *t* = 2.06, *p* = .039), Openness (*b* = – 0.0079, *t* = – 3.97, *p* < .001), and Conscientiousness (*b* = – 0.0048, *t* = – 2.37, *p* = .018), but not for Agreeableness (|*t*|< 1). Breaking down the interactions, in crowdsourced samples, there were positive effects of age on Openness (*b* = 0.0046, *t* = 2.58, *p* = .010) and Extraversion (*b* = 0.0131, *t* = 5.76, *p* < .001), while in panel recruitments, there was a negative effect of age on Openness (*b* = – 0.0034, *t* = – 3.05, *p* = .002) and no effect of age on Extraversion (|*t*|< 1). The other two significant interactions reflected differences in the magnitude but not the direction of the age effect. Effects of age on Neuroticism were more negative for crowdsourced samples (crowdsourced: *b* = – 0.0242, *t* = – 10.36, *p* < .001; panels: *b* = – 0.0189, *t* = – 13.51, *p* < .001), and effects of age on Conscientiousness were more positive for crowdsourced samples (crowdsourced: *b* = 0.0163, *t* = 8.87, *p* < .001; panels: *b* = 0.0114, *t* = 10.29, *p* < .001).

Within crowdsourced platforms, model-comparison ANOVAs indicated platform-specific effects for Neuroticism (*F*(4, 566) = 9.69, *p* < .001), Agreeableness (*F*(4, 566) = 5.77, *p* < .001), and Conscientiousness (*F*(4, 566) = 11.80, *p* < .001), but not Extraversion (*F*(4, 566) = 1.20, *p* = .31) or Openness (*F*(4, 566) < 1). The CR Toolkit sample, compared to the MTurk sample, showed lower Neuroticism (*b* = – 0.2415, *t* = – 2.44, *p* = .015) and higher Conscientiousness (*b* = 0.1952, *t* = 2.53, *p* = .012), but no difference in Agreeableness (*b* = 0.1037, *t* = 1.36, *p* = .17), at middle age. In contrast, relative to MTurk, the Prolific sample showed higher Neuroticism (*b* = 0.2655, *t* = 2.71, *p* = .007), lower Conscientiousness (*b* = – 0.2846, *t* = – 3.72, *p* < .001), and lower Agreeableness (*b* = – 0.1942, *t* = – 2.57, *p* = .010) at middle age.

Relative to MTurk, the CR Toolkit sample also showed less decrease in Neuroticism with age (*b* = 0.0152, *t* = 2.68, *p* = .008), less increase in Conscientiousness with age (*b* = – 0.0101, *t* = – 2.28, *p* = .023), and less increase in Agreeableness with age (*b* = – 0.0098, *t* = – 2.25, *p* = .025). The Prolific sample similarly showed less increase in Agreeableness with age than MTurk (*b* = – 0.0111, *t* = – 2.47, *p* = .014), but no difference from MTurk in effects of age on Neuroticism (*t* < 1) or Conscientiousness (*b* = – 0.0052, *t* = – 1.15, *p* = .25).

Within panel recruitments, model-comparison ANOVAs only indicated platform-specific effects for Agreeableness (*F*(4, 1178) = 2.47, *p* = .043). Agreeableness was marginally higher at middle age for Prime Panels compared to Lucid (*b* = 0.1036, *t* = 1.78, *p* = .076), but there was no difference between Prime Panels and Lucid in the effect of age (|*t*|< 1). In contrast, there was a greater increase in Agreeableness with age for Qualtrics Panels, relative to Lucid (*b* = 0.0054, *t* = 2.16, *p* = .031), but no difference between Qualtrics Panels and Lucid in Agreeableness at middle age (|*t*|< 1).

### Comparisons with GPIPP data

We compared the data from each of the six platforms to the GPIPP dataset, collected from a very large sample of unpaid online volunteers (Fig. 2, dashed lines). Detailed results and statistics are reported in the Supplement. Table 5 shows main effects indicating the direction of any differences between each platform and the comparison data at middle age, and the direction of any differences in the effect of age between each platform and the comparison data. Compared to the GPIPP sample, all platforms showed lower levels of Extraversion, and all platforms except Prolific showed lower Openness (with MTurk showing a marginal effect here), at middle age. Main effects for Agreeableness, Neuroticism, and Conscientiousness were more variable across platforms.

**Table 5:** Direction of effects on personality measures relative to GPIPP comparison sample (marginal effects are in parentheses)

	MTurk		CR tookit		Prolific		Lucid		Prime panels		Qualtrics panels	
	Main Effect	Age	Main Effect	Age	Main Effect	Age	Main Effect	Age	Main Effect	Age	Main Effect	Age
Extraversion	↓	↑	↓	↑	↓	↑	↓	—	↓	—	↓	—
Neuroticism	↓	↓	↓	↓	—	↓	—	↓	—	↓	↑	↓
Openness	(↓)	↑	↓	—	—	—	↓	↓	↓	—	↓	↓
Agreeableness	—	↑	↑	↑	↓	—	↓	↑	—	↑	↓	↑
Conscientiousness	↑	↑	↑	—	↓	↑	—	—	—	—	↓	↑

*Arrows* are defined as described above for Table 3

Regarding age differences, the effect of age on Extraversion was more positive in all three crowdsourced samples than in the GPIPP dataset but was not different from the GPIPP data in any of the three panel platforms. The effect of age on Neuroticism was more negative on all six platforms than in the comparison data, and the effects of age on Agreeableness were more positive than in the comparison data for all platforms except Prolific. Openness showed a more positive effect of age on MTurk than in the comparison data, but a more negative effect of age in samples from Lucid and Qualtrics, with other platforms not differing. Finally, effects of age on Conscientiousness were more positive than in the comparison data for MTurk, Prolific, and Qualtrics but matched the comparison data on other platforms.

### Political orientation measures

**Fig. 3:**
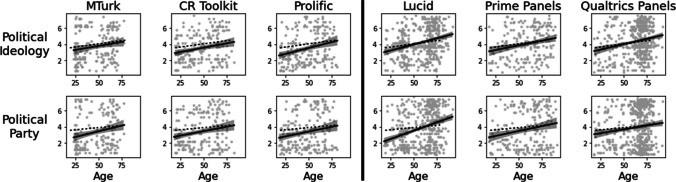
Relationships between age and political measures by platform, controlling for level of education, with *solid lines* showing linear regression effects and 95% confidence intervals shown within each platform. *Dashed lines* represent regression effects from the ANES 2020 sample, controlling for educational attainment

Within each of the six platforms, we found the expected increases with age in both conservative political ideology and Republican party affiliation (Fig. 3; Table 6). Combining across all platforms, there was an age-related increase on both conservative ideology (*b* = 0.0295, *t* = 12.02, *p* < .001) and Republican party affiliation (*b* = 0.0286, *t* = 9.98, *p* < .001).

**Table 6:** Effects of age on political preference measures within each platform, controlling for level of education

	MTurk			CR Toolkit			Prolific		
	*b*	*t*	Corrected *p*	*b*	*t*	Corrected *p*	*b*	*t*	Corrected *p*
Political Ideology	**0.0227**	**2.51**	.013*	**0.0217**	**3.16**	.004**	**0.0298**	**3.72**	.001***
Political Party	**0.0303**	**3.05**	.013*	**0.0221**	**2.79**	.013*	**0.0236**	**2.68**	.013*
	Lucid			Prime panels			Qualtrics panels		
	*b*	*t*	Corrected *p*	*b*	*t*	Corrected *p*	*b*	*t*	Corrected *p*
Political Ideology	**0.0301**	**6.04**	< .001***	**0.0227**	**3.31**	.003**	**0.0268**	**4.87**	< .001***
Political Party	**0.0409**	**6.97**	< .001***	**0.0245**	**2.97**	.013*	**0.0191**	**2.89**	.013*

Reported *p* values for each measure include a Bonferroni–Holm correction for multiple comparisons across the six platforms

### Comparisons across platforms

Comparing platform types, panel recruitments exhibited more conservative ideological orientation than crowdsourced samples at middle age (*b* = 0.3355, *t* = 3.28, *p* = .001), but there were no differences in political partisanship (*t* < 1). Neither ideology nor partisanship showed an interaction between platform type and age (*t* < 1). Furthermore, according to model-comparison ANOVAs, there were no platform-specific effects in ideology or partisanship in the crowdsourced samples (both *F* < 1), nor were there platform-specific effects in ideology (*F* < 1) or partisanship (*F*(4, 1176) = 1.88, *p* = .11) in the panel recruitments.

### Comparisons with ANES data

We used the 2020 ANES survey as a comparison dataset here, comparing the data from each of the six platforms to the ANES. Detailed results and statistics are reported in the Supplement. Table 7 shows the direction of any differences between each platform and the ANES sample. In all three crowdsourced samples, compared to the ANES, participants were both more ideologically liberal and more likely to identify as Democrats at middle age. Panel participants were also more likely to identify as Democrats than ANES participants at middle age, but these groups did not reliably differ in political ideology. Age effects generally did not differ significantly from the ANES sample, with the exception of Lucid, which showed a larger effect of age on both political ideology and partisan identity compared to the ANES sample.

**Table 7:** Direction of political orientation effects relative to ANES comparison sample (marginal effects are in parentheses)

	MTurk		CR toolkit		Prolific		Lucid		Prime panels		Qualtrics panels	
	Main Effect	Age	Main Effect	Age	Main Effect	Age	Main Effect	Age	Main Effect	Age	Main Effect	Age
Political Ideology	↓	—	↓	—	↓	(↑)	—	↑	(↓)	—	—	(↑)
Political Party	↓	—	↓	—	↓	—	↓	↑	↓	—	(↓)	—

*Arrows* are defined as described above for Table 3

## Discussion

This study aimed to determine whether expected age differences in cognitive performance, personality, and political orientation would be observed among participants recruited from paid online platforms. The most striking feature of these data is that age effects consistent with prior work were observed across all six platforms for the majority of measures examined. Regarding cognition, effects on vocabulary and digit symbol tests, which prior work has demonstrated show strong positive and negative age effects, respectively, were particularly consistent and robust. Paired associate memory additionally decreased with age, as would be expected based on prior work, in a manner that did not reliably differ between different platforms. Sustained attention similarly showed the expected change toward more cautious responding with age, with only a slight difference between platforms. Regarding personality, Neuroticism showed the expected negative correlation with age, while Agreeableness showed the expected positive correlation with age, and these effects were apparent across all platforms tested. Conscientiousness also showed a consistent positive correlation with age across all platforms, consistent with large samples of unpaid online participants in the GPIPP dataset (Soto et al., 2011; Srivastava et al., 2003), though not necessarily with in-person samples (cf., Graham et al., 2020). Finally, regarding political orientation, self-reported political ideology and partisan identity showed reliable conservative shifts with age, consistent with prior work and with the 2020 ANES survey data.

We also identified some broad differences between crowdsourced samples and panel recruitments. Although crowdsourced and panel platforms showed similar age differences in cognitive performance, participants in the crowdsourced samples scored better on all four cognitive tasks than those in the panel samples. These analyses compared the two platform types at the same midlife reference age, 48 years, ruling out the prospect that differences were driven by panel platforms skewing older on average than crowdsourced samples. It is not clear whether these differences reflect a motivational bias, in which participants in panels are less inclined to stay focused on online cognitive tasks, or a selection bias, in which participants in panels are truly less cognitively skilled. Further work would be needed to address these issues.

In contrast to the cognitive variables, age effects for many of the Big Five personality variables did differ between crowdsourced and panel samples. The two most notable differences were for Extraversion, which increased in age in crowdsourced samples but showed no effect in panel samples, and for Openness, which increased with age in crowdsourced samples and decreased in panel samples. The increase in Extraversion with age in crowdsourced samples is particularly anomalous, while the lack of a reliable effect of age in panel samples is more consistent with prior work. The prior literature is less clear regarding the effects of age on Openness. On one hand, the more recent published study from the GPIPP dataset (Soto et al., 2011), and the larger GPIPP sample covering data up to 2015 that we used as a comparison sample, both showed positive effects of age on Openness. On the other hand, many other studies have observed a negative effect of age on Openness, including the earlier subset of the GPIPP data (Srivastava et al., 2003), various in-person samples (e.g., Donnellan & Lucas, 2008; Terracciano et al., 2005), and meta-analyses of longitudinal studies (Graham et al., 2020; Roberts et al., 2006). On balance, we would tentatively conclude that a negative relationship between age and Openness is more likely the ground truth. Thus, the results for both Extraversion and Openness would suggest that, relative to crowdsourced samples, panels may be a more representative cross section of the population for examining cross-sectional effects of age on personality.

Beyond these broad differences between crowdsourced and panel platforms, we also found that MTurk was a notable outlier among crowdsourced platforms. The MTurk sample showed poorer vocabulary performance at middle age than either CR Toolkit or Prolific, and MTurk also showed at least marginally poorer performance than CR Toolkit on sustained attention and digit symbol coding at middle age. The MTurk sample also exhibited a different pattern of age effects on cognitive tasks. One clear deviation was that older adults on MTurk showed better sustained attention than younger adults on MTurk. MTurk was the only platform on which participants showed a reliable increase with age in gradCPT d’ and the only platform to show a more positive effect of age on this task than the Testmybrain comparison sample. Additionally, MTurk participants showed a greater increase in vocabulary performance with age, and less of decrease in digit symbol coding performance with age, than the other two crowdsourced platforms; both effects suggest relatively higher cognitive performance in older adults on MTurk. These data alone do not clarify whether these differences are due to older adults, younger adults, or both, on MTurk. However, as discussed above, others have seen poorer performance and higher rates of inattentive responding on MTurk compared to other platforms (see also Kay, 2025), and both CloudResearch and Prolific actively work to maintain high-quality participant pools. Since more of the participants on these platforms are younger, studies that do not stratify by age recruit largely young adults. Thus, these interactions between crowdsourced platform and age are likely to be driven at least in part by relatively poorer performance among young adult participants. In addition, recruiting an age-stratified sample was more expensive on MTurk than on the other crowdsourced platforms, as MTurk required both a full 40% platform fee plus a separate fee to stratify by age, rather than the discounted 20% fee applied otherwise, including in CR Toolkit. All of these factors suggest that researchers should be cautious about using MTurk to conduct online studies of cognitive aging.

One area where all online platforms deviated from a population sample was in political orientation. Participants across all platforms were at least marginally more likely to identify as Democrats than the ANES population sample, and participants on the three crowdsourced platforms were also more likely to identify as ideologically liberal than the ANES sample. This extends prior findings from the early days of online data collection that online participants tend to be more liberal than the population at large (e.g., Berinsky et al., 2012). Still, participants on all platforms showed the expected relationship between age and increasing conservatism.

One potential limitation of our study stems from differences between samples collected on crowdsourced platforms and those collected via panels. In particular, all three panel services were able to approach or meet our desired quota of 30 participants age 80 + . None of the crowdsourcing platforms even allowed researchers to request participants in this oldest age group, and indeed, very few such participants completed the study on those platforms. In part because of this, the mean age was over ten years higher for panels. Additionally, two of the panels (Lucid and Qualtrics Panels) greatly oversampled participants aged 60–80. Still, all main effects of platform type or of platform are estimated at the same reference age for all analyses, which should control for these differences. Additionally, all reported effects of age are linear, and we believe that all samples represent the adult lifespan sufficiently well to detect linear effects on cognition, personality, and political orientation. We do note, however, that panels are likely to be a better choice for researchers aiming to examine changes that occur across the older adult age range, e.g., comparing those age 60–70 years to age 75 and above.

Ultimately, these results tell a broadly optimistic story regarding the ability to use online participant recruitment to test questions related to cognitive aging. All of the tested platforms could provide substantial samples of individuals across the lifespan at a lower cost in money and time than a comparable in-person sample. The data from participants on those platforms was also generally consistent with age differences previously established in in-person samples. Thus, we conclude that age differences can be assessed using online participant recruitment, though different platforms may be better suited for different questions. Specifically, filtered crowdsourced samples, such as CR Toolkit and Prolific, may be slightly better suited to cognitive studies, whereas panel samples may be slightly better suited for studies or personality or political orientation where population representativeness is important.

## Supplementary Information

Below is the link to the electronic supplementary material.

Supplementary file1 (PDF 149 kb)

## Data Availability

All data that were collected as part of this project are shared on OSF at https://osf.io/2qd7x/. The tasks and comparison data from TestMyBrain/Many Brains Project, and the comparison data from GPIPP and ANES, are not shared in our OSF repository because we are not the creators/owners of those materials.

## Code availability

Analysis code is shared in the OSF repository at https://osf.io/2qd7x/. Specifically, an input file and script that generate all statistical analyses in the paper, except those involving external comparison data, are shared. Input files and scripts to generate versions of the figures without comparison data are also shared. Finally, we provide the full data processing script for transparency, but without the input files needed to run it, for privacy reasons (i.e., this script uses subject IDs from the platforms to unite different raw data sources) and due to the data ownership concerns on external comparison datasets noted above.

## Declarations

### Open practices statement

See above for availability of data and materials and code availability. This study was not preregistered.
