# Supplementary material for: Comparing age differences in cognition, personality, and political orientation across six online recruitment platforms
Source: Behav Res Methods. 2026 Apr 28;58(6):144. doi: 10.3758/s13428-026-03027-8 (PMC13124840; doi:10.3758/s13428-026-03027-8)
Supplement: Supplementary file 1 — Supplementary file1 (PDF 149 kb) [file 13428_2026_3027_MOESM1_ESM.pdf]

“Comparing age differences in cognition, personality, and political orientation across six online recruitment platforms”

Supplemental Results

In regressions run to compare data from each of the six platforms to comparison data for that measure (as the reference condition), the comparison dataset for a given task was combined with the data that we collected for that task. Twelve dummy regressors were included to reflect platform main effects and each platform’s interaction with age. Main effects of age and education were also modeled. These regressions show that at baseline, i.e., based on the comparison data from Testmybrain, we see the expected positive effect of age on vocabulary (b = 0.0067, t = 90.48, p < .001) and negative effects of age on digit symbol coding (b = -0.3899, t = -35.44, p < .001) and paired-associate memory (b = -0.0014, t = -8.36, p < .001). There was also a positive effect of age on gradCPT d’ (b = 0.0042, t = 9.96, p < .001), and age predicted a more conservative gradCPT criterion (b = -0.0060, t = -24.84, p < .001). Higher level of education also predicted better performance on vocabulary (b = 0.0100, t = 22.28, p < .001), digit symbol coding (b = 0.5726, t = 11.37, p < .001), paired-associate memory (b = 0.0136, t = 17.40, p < .001), and gradCPT d’ (b = 0.0438, t = 21.22, p < .001), and a more conservative criterion (b = -0.0071, t = -5.99, p < .001). Statistics representing main effects of each platform, and interactions between platform and age, are shown in Supplemental Table 3.

Similar regressions were run to compare personality measures to comparison data from the GPIPP dataset incorporating all data up to 03/25/2015. All participants who reported living in the United States and ranging in age from 18 to 85 were included, encompassing 2,669,696 data points. At baseline, we see negative effects of age on extraversion (b = -0.0002, t = -5.04, p < .001) and on neuroticism (b = -0.0054, t = -118.24, p < .001). We see positive effects of age on agreeableness (b = 0.0041, t = 111.45, p < .001), openness (b = 0.0023, t = 61.18, p < .001), and conscientiousness (b = 0.0093, t = 243.20, p < .001). Education was associated with positive

For Review Only

effects on extraversion (b = 0.0015, $t = 5.27$, $p < .001$), openness (b = 0.0359, $t = 164.65$, $p < .001$), and conscientiousness (b = 0.0222, $t = 98.21$, $p < .001$), and with negative effects on neuroticism (b = -0.0099, $t = -36.36$, $p < .001$) and agreeableness (b = -0.0095, $t = -43.25$, $p < .001$). Statistics representing main effects of each platform, and interactions between platform and age, are shown in Supplemental Table 4.

Similar regressions were run to compare data on political orientation and ideology to comparison data provided in the 2020 ANES dataset. For political ideology, the baseline ANES sample showed a strong positive main effect of age (b = 0.0176, $t = 15.20$, $p < .001$) and a strong negative main effect of education (b = -0.1189, $t = -14.95$, $p < .001$), indicating that older participants and those with lower levels of education tended to be more ideologically conservative. With political party identity as the outcome measure, the baseline ANES sample again showed a positive effect of age (b = 0.0137, $t = 8.78$, $p < .001$) and a negative effect of education (b = -0.1307, $t = -12.23$, $p < .001$), indicating that older and less well-educated participants were also more likely to identify as Republican. Statistics representing main effects of each platform, and interactions between platform and age, are shown in Supplemental Table 5.

For Review Only

Supplemental Table 1. Cost of data collection on each platform.

	Cost
Amazon MTurk	$1,270.40
CR Toolkit	$1,209.83
Prolific	$1,120.00
Lucid	$871.50[a]
Prime Panels	$1,009.33
Qualtrics Panels	$2,102.50[b]

[a] Minimum commitment (for current and future studies) = $1,500

[b] Includes $1,000 required fee for integration with non-Qualtrics data collection platform

For Review Only

Supplemental Table 2. Coding of educational attainment in years based on categorical responses in each dataset

**Primary data**		**Testmybrain**		**GSIPP**		**ANES**	
**Category**	**Years**	**Category**	**Years**	**Category**	**Years**	**Category**	**Years**
Did not finish high school	11	Primary	5	Currently in high school	11	8th grade or less	8
High school	12	Middle school	8	Did not finish high school	11	Completed between 9-12 grades, no diploma	11
Some college	13	High school	12	Completed high school	12	High school diploma	12
Associate's Degree	14	Some college	13	Currently in college	13	High school diploma with nonacademic training	13
Bachelor's Degree	16	Technical	13	Some college	13	Some college or Associate's degree	13
Master's Degree	18	College	16	Completed Bachelor's degree	16	Bachelor's degree	16
Doctoral Degree	20	Graduate Degree	18	Currently in graduate school	16	Advanced degree	18
				Graduate or professional degree	18		

Supplemental Table 3. Main effects and age differences relative to Testmybrain comparison data for each cognitive measure.

	Vocabulary					
	Main Effect			Age		
	b	t	p	b	t	p
**MTurk**	**-0.1196**	**-8.13**	**< .001*** **	0.0010	1.16	.25
**CR Toolkit**	**-0.0321**	**-2.31**	**.021***	**-0.0034**	**-4.49**	**< .001*** **
**Prolific**	**-0.0518**	**-3.78**	**< .001*** **	**-0.0027**	**-3.43**	**.001** **
**Lucid**	**-0.1874**	**-17.54**	**< .001*** **	0	-0.16	.87
**Prime Panels**	**-0.1343**	**-9.02**	**< .001*** **	**-0.0023**	**-3.32**	**.001** **
**Qualtrics Panels**	**-0.1823**	**-15.21**	**< .001*** **	-0.0004	-0.73	.47

	Digit Symbol Coding					
	Main Effect			Age		
	b	t	p	b	t	p
**MTurk**	-0.3910	-0.42	.68	**0.1134**	**2.04**	**.041***
**CR Toolkit**	**2.1520**	**2.41**	**.016***	**-0.1304**	**-2.68**	**.007** **
**Prolific**	0.3283	0.37	.71	**-0.1060**	**-2.09**	**.037***
**Lucid**	**-6.9191**	**-9.90**	**< .001*** **	-0.0328	-0.92	.36
**Prime Panels**	**-5.4418**	**-5.70**	**< .001*** **	**-0.1032**	**-2.32**	**.020***
**Qualtrics Panels**	**-6.6986**	**-8.61**	**< .001*** **	-0.0164	-0.45	.65

	Paired Assoc Memory					
	Main Effect			Age		
	b	t	p	b	t	p
**MTurk**	**-0.1046**	**-6.92**	**< .001*** **	0.0011	1.29	.20
**CR Toolkit**	**-0.0838**	**-5.85**	**< .001*** **	0.0003	0.35	.73
**Prolific**	**-0.0986**	**-6.98**	**< .001*** **	-0.0010	-1.29	.20
**Lucid**	**-0.1673**	**-14.89**	**< .001*** **	-0.0005	-0.80	.43
**Prime Panels**	**-0.1691**	**-11.05**	**< .001*** **	-0.0009	-1.20	.23
**Qualtrics Panels**	**-0.1670**	**-13.37**	**< .001*** **	-0.0001	-0.17	.87

	GradCPT d'					
	Main Effect			Age		
	b	t	p	b	t	p
**MTurk**	**-0.1832**	**-3.09**	**.002** **	**0.0186**	**5.31**	**< .001*** **
**CR Toolkit**	0.0693	1.24	.22	-0.0027	-0.88	.38
**Prolific**	**-0.2370**	**-4.29**	**< .001*** **	-0.0043	-1.33	.18
**Lucid**	**-0.6865**	**-15.83**	**< .001*** **	-0.0015	-0.66	.51
**Prime Panels**	**-0.6732**	**-11.20**	**< .001*** **	-0.0051	-1.84	.066 ~
**Qualtrics Panels**	**-0.7604**	**-15.67**	**< .001*** **	0.0022	0.98	.33

For Review Only

	GradCPT criterion					
	Main Effect			Age		
	b	t	p	b	t	p
**MTurk**	**0.1260**	**3.72**	**< .001*****	0.0037	1.87	.062 ~
**CR Toolkit**	**0.1702**	**5.32**	**< .001*****	-0.0002	-0.14	.89
**Prolific**	**0.1312**	**4.16**	**< .001*****	0.0001	0.06	.95
**Lucid**	**-0.0523**	**-2.11**	**.035***	0.0023	1.86	.063 ~
**Prime Panels**	-0.0519	-1.51	.13	0.0029	1.83	.067 ~
**Qualtrics Panels**	**-0.1478**	**-5.34**	**< .001*****	**0.0071**	**5.49**	**< .001*****

For Review Only

Supplemental Table 4. Main effects and age differences relative to GPIPP comparison data for each personality measure.

	**Extraversion**					
	**Main Effect**			**Age**		
	b	t	p	b	t	p
**MTurk**	**-0.3247**	**-5.09**	**< .001*** **	**0.0184**	**4.87**	**< .001*** **
**CR Toolkit**	**-0.3889**	**-6.45**	**< .001*** **	**0.0143**	**4.33**	**< .001*** **
**Prolific**	**-0.4028**	**-6.78**	**< .001*** **	**0.0088**	**2.53**	**.011***
**Lucid**	**-0.2345**	**-5.08**	**< .001*** **	-0.0016	-0.67	.50
**Prime Panels**	**-0.3415**	**-5.28**	**< .001*** **	0.0001	0.02	.99
**Qualtrics Panels**	**-0.3181**	**-6.13**	**< .001*** **	0.0001	0.04	.97

For Review Only

	**Neuroticism**					
	**Main Effect**			**Age**		
	b	t	p	b	t	p
**MTurk**	**-0.2075**	**-3.34**	**.001** **	**-0.0245**	**-6.64**	**< .001*** **
**CR Toolkit**	**-0.4378**	**-7.46**	**< .001*** **	**-0.0100**	**-3.12**	**.002** **
**Prolific**	0.0480	0.83	.41	**-0.0237**	**-7.03**	**< .001*** **
**Lucid**	0.0158	0.35	.73	**-0.0126**	**-5.48**	**< .001*** **
**Prime Panels**	-0.0324	-0.52	.61	**-0.0100**	**-3.42**	**.001** **
**Qualtrics Panels**	**0.1053**	**2.09**	**.037***	**-0.0171**	**-7.25**	**< .001*** **

	**Openness**					
	**Main Effect**			**Age**		
	b	t	p	b	t	p
**MTurk**	-0.0921	-1.84	.066 ~	**0.0064**	**2.15**	**.032***
**CR Toolkit**	**-0.0933**	**-1.98**	**.048***	0.0013	0.52	.60
**Prolific**	-0.0749	-1.61	.11	0.0000	0.01	.99
**Lucid**	**-0.2437**	**-6.74**	**< .001*** **	**-0.0056**	**-3.05**	**.002***
**Prime Panels**	**-0.3591**	**-7.09**	**< .001*** **	-0.0023	-0.98	.33
**Qualtrics Panels**	**-0.2871**	**-7.07**	**< .001*** **	**-0.0074**	**-3.89**	**< .001*** **

	**Agreeableness**					
	**Main Effect**			**Age**		
	b	t	p	b	t	p
**MTurk**	0.0041	0.08	.93	**0.0149**	**4.99**	**< .001*** **
**CR Toolkit**	**0.1017**	**2.15**	**.032***	**0.0054**	**2.09**	**.036***
**Prolific**	**-0.1847**	**-3.95**	**< .001*** **	0.0044	1.60	.11
**Lucid**	**-0.1608**	**-4.43**	**< .001*** **	**0.0053**	**2.83**	**.005** **
**Prime Panels**	-0.0535	-1.05	.29	**0.0047**	**2.00**	**.045***
**Qualtrics Panels**	**-0.1918**	**-4.70**	**< .001*** **	**0.0106**	**5.53**	**< .001*** **

	Conscientiousness					
	Main Effect			Age		
	b	t	p	b	t	p
**MTurk**	**0.1440**	**2.78**	**.005**	**0.0131**	**4.26**	**< .001*****
**CR Toolkit**	**0.3526**	**7.22**	**< .001*****	0.0021	0.79	.43
**Prolific**	**-0.1524**	**-3.17**	**< .001*****	**0.0067**	**2.38**	**.018***
**Lucid**	-0.0294	-0.79	.43	0.0007	0.36	.72
**Prime Panels**	-0.0428	-0.82	.41	0.0002	0.09	.93
**Qualtrics Panels**	**-0.1398**	**-3.33**	**.001****	**0.0053**	**2.69**	**.007****

Supplemental Table 5. Main effects and age differences relative to ANES for political measures.

	Political Ideology					
	Main Effect			Age		
	b	t	p	b	t	p
**MTurk**	**-0.3188**	**-2.52**	**.012***	0.0020	0.27	.79
**CR Toolkit**	**-0.5381**	**-4.51**	**< .001*****	0.0041	0.62	.54
**Prolific**	**-0.5977**	**-5.06**	**< .001*****	0.0117	1.71	.088 ~
**Lucid**	-0.1544	-1.64	.10	**0.0128**	**2.66**	**.008****
**Prime Panels**	-0.2556	-1.93	.054 ~	0.0056	0.90	.37
**Qualtrics Panels**	-0.1163	-1.09	.28	0.0091	1.80	.071 ~

	Political Party					
	Main Effect			Age		
	b	t	p	b	t	p
**MTurk**	**-0.5232**	**-3.07**	**.002****	0.0146	1.45	.15
**CR Toolkit**	**-0.4904**	**-3.06**	**.002****	0.0085	0.97	.34
**Prolific**	**-0.5539**	**-3.48**	**< .001*****	0.0101	1.09	.27
**Lucid**	**-0.4718**	**-3.78**	**< .001*****	**0.0276**	**4.32**	**< .001*****
**Prime Panels**	**-0.4809**	**-2.80**	**.005****	0.0105	1.31	.19
**Qualtrics Panels**	-0.2593	-1.87	.062 ~	0.0054	0.83	.41

For Review Only
